# Influence of elemental sulfur on cadmium bioavailability, microbial community in paddy soil and Cd accumulation in rice plants

**DOI:** 10.1038/s41598-021-91003-x

**Published:** 2021-06-01

**Authors:** Lijuan Sun, Ke Song, Lizheng Shi, Dechao Duan, Hong Zhang, Yafei Sun, Qin Qin, Yong Xue

**Affiliations:** 1 ECO-Environment Protection Research Institute, Shanghai Academy of Agricultural Sciences, Shanghai, 201403 China; 2 Shanghai Environmental Protection Monitoring Station of Agriculture, Shanghai, 201403 China; 3 Ministry of Ecology and Environmental, South China Institute of Environmental Sciences, No. 16-18, Ruihe Road, Huangpu District, Guangzhou, 510530 Guangdong China; 4 Zhejiang Towards Environment Co., Ltd, Hangzhou, 310012 China

**Keywords:** Environmental sciences, Biogeochemistry

## Abstract

Cadmium (Cd) is highly toxic to living organisms and the contamination of Cd in paddy soil in China has received much attention. In the present study, by conducting pot experiment, the influence of S fertilizer (S^0^) on rice growth, iron plaque formation, Cd accumulation in rice plants and bacterial community in rice rhizosphere soil was investigated. The biomass of rice plants was significantly increased by S^0^ addition (19.5–73.6%). The addition of S^0^ increased the formation of iron plaque by 24.3–45.8%, meanwhile the amount of Cd sequestered on iron plaque increased. In soil treated with 5 mg/kg Cd, addition of 0.2 g/kg S^0^ decreased the diffusive gradients in thin films (DGT) extractable Cd by 60.0%. The application of S^0^ significantly decreased the concentration of Cd in rice grain by 12.1% (0.1 g/kg) and 36.6% (0.2 g/kg) respectively. The addition of S^0^ significantly increased the ratio of *Acidobacteria*, *Bacteroidetes* in rice rhizosphere soil. Meanwhile, the ratio of *Planctomycetes* and *Chloroflexi* decreased. The results indicated that promoting Fe- and S-reducing and residue decomposition bacterial in the rhizosphere by S^0^ may be one biological reason for reducing Cd risk in the soil-rice system.

## Introduction

Cadmium (Cd) is well known to be highly toxic to living organisms. Due to its comparatively high mobility in soil–plant system, cadmium can be easily taken up by crops and subsequently translocated to the edible organs^1^. Long term exposure to Cd contaminated food will cause chronic toxicity to human organs, leading to diseases like cancer, arthrophlogosis, renal tubular necrosis, Itai-Itai et al.^2^ About 2.786 × 10^9^ m^2^ of agricultural soils were polluted with Cd in China^3^. Therefore, exploring efficiency way to reduce the amount and mobility of Cd in soil or to limit its uptake and accumulation in crops is very necessary.

The toxicity of Cd to organisms is largely determined by its bioavailability in soil. Soil chemical properties like pH, redox potential (Eh), content of organic matter, cation exchange capacity and content of clays, Fe, and Mn oxides are the main environmental factors affecting Cd bioavailability. Under reduced flooding condition, part of the available Cd in soil can transform into CdS that generally have low solubility product constants (K_sp_ = 10^–27^)^4^. Rice is mostly grown under flooding conditions and formation of CdS in paddy soil is an significant way to reduce the bioavailability of Cd^5^, thus providing implication of using sulfur to regulate the mobility of Cd in paddy soil. Sulfur is an essential macronutrient for plant, application of sulfur fertilizer in soil has received much attention recent years due to the frequent sulfur deficiency in soil systems^6,7^.

Besides the reduction of Cd bioavailability in paddy soil, the sequestration of Cd by iron plaque formed on the surface of rice roots is another way to decrease the uptake of Cd by rice plants. Iron plaque is commonly formed due to the oxidation of ferrous to ferric iron and the precipitation of ferric oxide on the rice root surfaces^8^. The formation of iron plaque is influenced by many physical–chemical characteristics of soils and/or sediments, e.g., texture, organic matter, pH, reduction potential (Eh), water management, Fe and Mn fertilization, phosphorus, and sulfur (S) supply^9^. Our previous study found that sulfur fertilization (less than 500 mg/kg) promoted the formation of iron plaque on rice roots surface, however, excessive S can be toxic to rice roots leading to root rot, as well as reducing iron plaque formation^10^. Low amount of sulfur fertilizer is supposed to promote iron plaque formation, thus reducing the uptake of heavy metal by rice roots. Application of sulfur fertilizer can be one way to reduce Cd uptake by rice plants due to the sequestration of Cd by the enhanced formation of iron plaque^11^. Sulfur has the potential of reducing Cd bioavailability by formation of CdS and enhancing Cd sequestration by inducing iron plaque formation in paddy soil, thus making sulfur fertilizer a promising candidate for remediation of Cd contaminated paddy soil. However, the interactions between sulfur fertilizer, iron plaque formation, Cd bioavailability and Cd accumulation in rice are still unclear.

Apart from physical–chemical factors, microbial is another important factor that influences the biogeochemical process of pollutant or nutrient element in soil. The biogeochemical behavior of sulfur in soil is closely related to soil microorganisms, including sulfur oxidizing bacteria (SOB) and sulfur reducing bacteria (SRB). Sulfur reducing bacteria are ubiquitous in paddy soil and they are also closely related to the speciation transformation of heavy metal like Fe and As^12^. The formation of sulfide by reduction of sulfur is of significant importance for the formation of metal sulfide, which controls the bioavailability and mobility of heavy metal. Sulfur fertilizer application has been found to significantly change the rhizosphere microbial community and increased the sulfur oxidizing bacteria^13^. However, the biological mechanism of sulfur fertilizer on the bioavailability and mobility of Cd in paddy soil is still unclear.

In the present study, by conducting pot experiment, the effect of sulfur fertilizer on Cd speciation transformation and bioavailability in paddy soil as well as microbial community in rice rhizosphere soil was studied. The aim of the present study were to: (1) evaluate the effect of sulfur fertilizer on Cd bioavailability and accumulation in rice plants; (2) investigate the changes in microbial communities in Cd contaminated paddy soil with the addition of sulfur fertilizer.

## Result

### Rice plant growth and Cd accumulation

**Figure 1:**
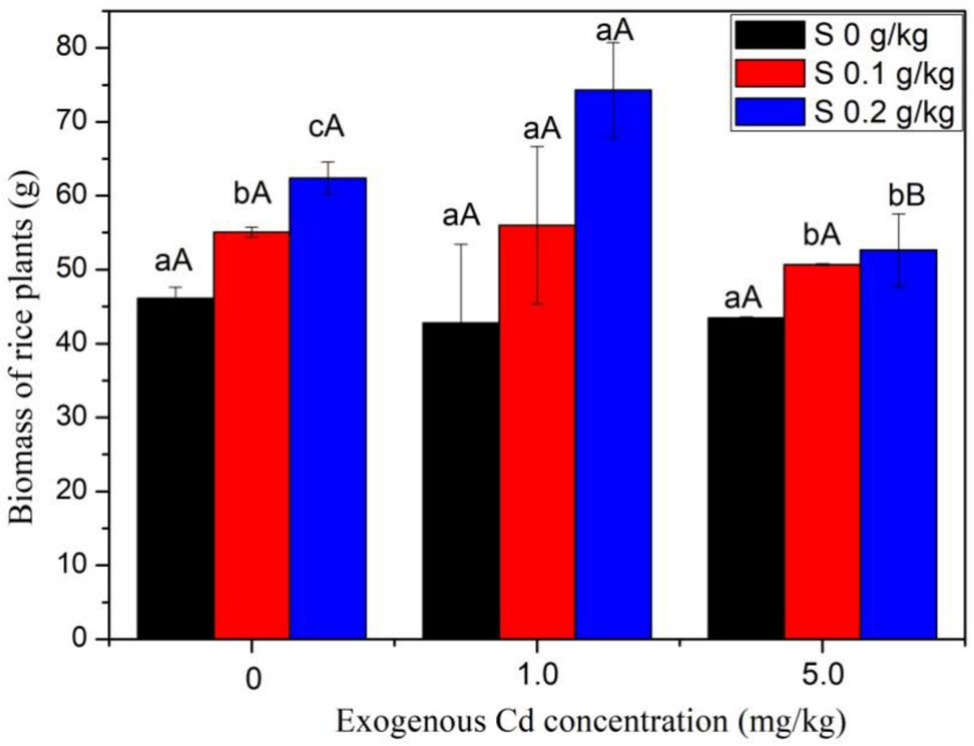
The effect of S application on biomass of rice plant. Different lowercase letters within biomass for rice indicates a significant difference among different S levels at *P* < 0.05. Different capital letters within biomass for rice indicates a significant difference among different Cd treatments at *P* < 0.05.

The application of S^0^ promoted the growth of rice plants (Fig. 1). In soil without Cd, the biomass of rice plants in S^0^ treatments increased by 19.5–35.3%. In soil treated with Cd, the biomass of rice plants in 0.2 g/kg S^0^ treatments was significantly (*P* < 0.05) higher than that in the treatment without S^0^. Meanwhile, among all the treatments of 0.2 g/kg S^0^, the biomass of rice in soil treated with 1 mg/kg Cd was significant higher than those in soil treated without Cd and in soil treated with 5 mg/kg Cd. Although not significant, a trend of increase occurred in 0.1 g/kg S^0^ treatments when compared with the treatment without S^0^.

The concentration of Cd in different rice tissues was shown in Table 1. In soil treated with 1 mg/kg Cd, application of 0.1 g/kg S^0^ significantly decreased the concentration of Cd in rice root by 40.11% but no significant difference was found in leaf and grain. In soil treated with 5 mg/kg Cd, the accumulation of Cd in different parts of rice followed the descending order: root > stem > leaf > grain. The addition of S^0^ significantly decreased the concentration of Cd in rice root, stem and leaf. When compared with the control, the application of 0.1 g/kg S^0^ significantly decreased the concentration of Cd in rice stem by 21.94% and leaf by 13.76% (*P* < 0.05) respectively. The application of 0.2 g/kg S^0^ significantly decreased the concentration of Cd in rice root by 50.79%, stem by 26.45%, leaf by 48.17% and grain by 26.8%. In soil treated without Cd, application of S^0^ significantly decreased the concentration of Cd in rice root and grain, and no significant difference was found in leaf, a trend of increase was found in stem.

**Table 1:** The effect of sulfur application on Cd concentration in different tissues (mean ± S.E., n = 3).

S level (g/kg)	Root			Stem			Leaf			**Grain**		
	Cd 0 mg/kg	Cd 1 mg/kg	Cd 5 mg/kg	Cd 0 mg/kg	Cd 1 mg/kg	Cd 5 mg/kg	Cd 0 mg/kg	Cd 1 mg/kg	Cd 5 mg/kg	**Cd** **0 mg/kg**	**Cd** **1 mg/kg**	**Cd** **5 mg/kg**
0	0.28 ± 0.06 aA	1.77 ± 0.21 aB	8.25 ± 0.56 aC	0.06 ± 0.02 aA	1.21 ± 0.16 aB	3.1 ± 0.12 aC	0.43 ± 0.06 aA	0.88 ± 0.10 aB	2.18 ± 0.10 aC	0.06 ± 0.01 aA	0.17 ± 0.04 aB	0.41 ± 0.06 aC
0.1	0.25 ± 0.08 aA	1.06 ± 0.07 bB	7.14 ± 0.22 aC	0.55 ± 0.01 bA	1.49 ± 0.09 bB	2.42 ± 0.03 bC	0.35 ± 0.05 aA	0.84 ± 0.06 aB	1.88 ± 0.08 bC	0.05 ± 0.00 aA	0.18 ± 0.04 aB	0.34 ± 0.03 abC
0.2	0.19 ± 0.02 bA	1.35 ± 0.12 aB	4.06 ± 0.41 bC	0.42 ± 0.05 cA	1.42 ± 0.08 aB	2.28 ± 2.28 bC	0.36 ± 0.03 aA	0.95 ± 0.10 aB	1.13 ± 0.11 cC	0.03 ± 0.00 bA	0.18 ± 0.04 aB	0.30 ± 0.04 bC
**Analysis of variance**												
Cd levels	1484.13***			1770.24***			756.61***			1485.68***		
S levels	83.664***			5.34*			49.02***			83.87***		
Cd × S levels	66.91***			63.36***			55.69***			66.92***		

Different lowercase letters within Cd in root, stem, leaf, grain under the same Cd treatment indicates a significant difference among different S levels at *P* < 0.05.

Different capital letters within Cd in root, stem, leaf, grain under the same S treatment indicates a significant difference among different Cd treatment at *P* < 0.05.

Analysis of variance *P < 0.05, **P < 0.01, ***P < 0.001.

### Iron plaque formation and Cd accumulation in iron plaque

The concentration of ACA-extractable Fe and Cd in rice iron plaque was shown in Table 2. In soil treated without sulfur, the concentration of ACA-extractable Fe decreased with the addition of Cd, which meant the formation of iron plaque decreased with Cd stress. In soil treated with Cd, When compared with the control, the application of 0.1 g/kg S^0^, 0.2 g/kg S^0^ increased the concentration of ACA-extractable Fe by 35.1%, 45.8% respectively in soil treated with 1 mg/kg Cd and 24.3%, 31.3% respectively in soil treated with 5 mg/kg Cd. In soil treated with 1 mg/kg Cd, a trend of increase was found in the concentration of ACA-extractable Cd with the application of S^0^. In soil treated with 5 mg/kg Cd, the concentration of ACA-extractable Cd was significantly increased by the application of 0.2 g/kg S^0^ (*P* < 0.05). The concentration of ACA-extractable Cd was significantly increased by Cd addition. When compared with soil treated without Cd, the concentration of ACA-extractable Cd in soil treated with 1 mg/kg Cd and 5 mg/kg Cd increased by 146.3–160.4% and 210.9–310.7% respectively.

**Table 2:** The effect of sulfur application on ACA-extractable Fe and Cd concentration in rice iron plaque (mean ± S.E., n = 3).

S level (g/kg)	Cd (0 mg/kg)	Cd (1 mg/kg)	Cd (5 mg/kg)	Cd (0 mg/kg)	Cd (1 mg/kg)	Cd (5 mg/kg)
	Fe (mg/kg)	Fe (mg/kg)	Fe (mg/kg)	Cd (mg/kg)	Cd (mg/kg)	Cd (mg/kg)
0	15,653.21 ± 1629.24 aA	13,352.38 ± 3973.05 aA	13,282.61 ± 693.03 aB	2.01 ± 0.38 aA	4.95 ± 0.54 aB	6.25 ± 0.48 aB
0.1	15,511.87 ± 1696.18 aA	18,043.01 ± 691.26 bB	16,510.99 ± 1517.84 aAB	2.03 ± 0.51 aA	5.03 ± 1.02 aB	8.65 ± 1.96 abC
0.2	16,488.80 ± 1106.42 aA	19,468.68 ± 244.96 bB	17,444.21 ± 614.69 bA	2.25 ± 0.20 aA	5.86 ± 0.74 aB	9.24 ± 0.82 bC
**Analysis of variance**						
Cd levels	2.65			194.92***		
S levels	21.85***			10.58***		
Cd × S levels	4.48*			4.88**		

Different lowercase letters within Cd in root, stem, leaf, grain under the same Cd treatment indicates a significant difference among different S levels at *P* < 0.05.

Different capital letters within Cd in root, stem, leaf, grain under the same S treatment indicates a significant difference among different Cd treatment at *P* < 0.05.

Analysis of variance *P < 0.05, **P < 0.01, ***P < 0.001.

### Diffusive gradients in thin films (DGT) extractable Cd in the paddy soil of rice rhizosphere

**Figure 2:**
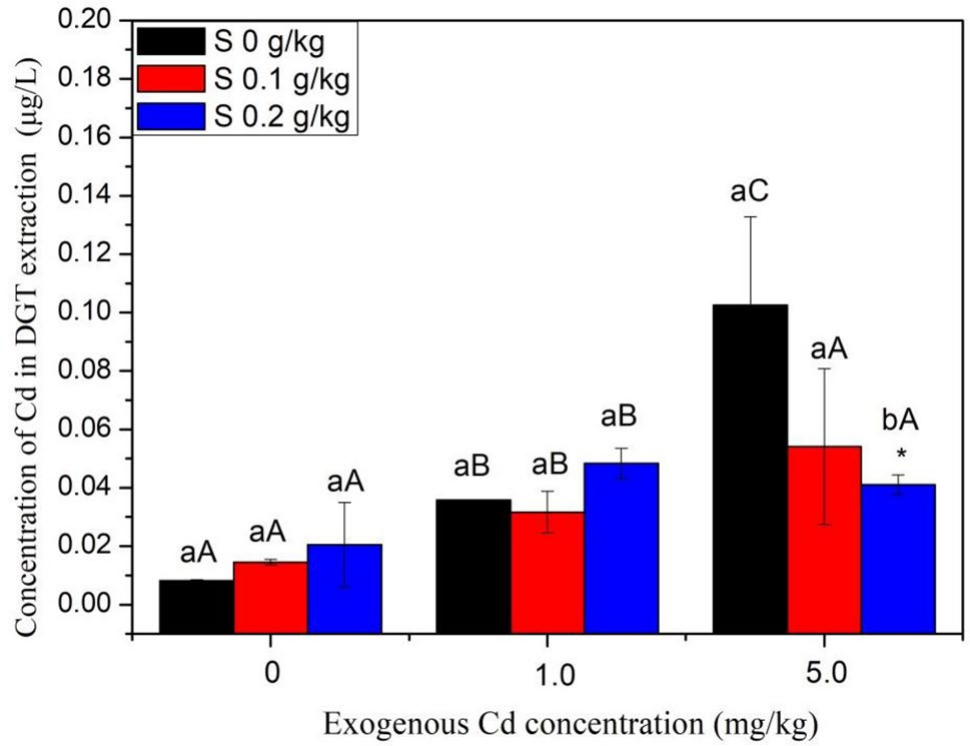
Concentration of DGT-extractable Cd in different rice rhizosphere soil. Different lowercase letters within biomass for rice indicates a significant difference among different S levels at *P* < 0.05. Different capital letters within biomass for rice indicates a significant difference among different Cd treatments at *P* < 0.05.

Diffusive gradients in thin films (DGT) technique is based on Fick’s first diffusion law, which is widely used to in situ measure content of the biological effective metals in environmental media^14^. The concentration of Cd extracted by DGT was shown in Fig. 2. In soil treated without or with 1 mg/kg Cd, no significant difference was found among different sulfur treatments. However, in soil treated with 5 mg/kg Cd, addition of 0.2 g/kg S^0^ significantly (*P* < 0.05) decreased the DGT extractable Cd by 60.0%. Although not significantly, addition of 0.1 g/kg S^0^ decreased the DGT extractable Cd by 47.2%.

### Bacterial community analysis

A total of 888,946 bacterial reads were obtained from the rhizosphere soil samples and the average sequence length was 454 bp. Read classification revealed a total of 66,200 operational taxonomic units (OTUs). The results of α diversity indices such as ACE, Chao1, Shannon and Simpson showed no significant differences among the rhizosphere soil treated with 5 mg/kg Cd with different sulfur treatments (Table 3).

**Table 3:** Alpha diversity of bacterial communities in rhizosphere soil treated with 5 mg/kg Cd under different S fertilization treatments. Italic number indicates the standard deviation.

Treatments	Reads	OTU	ACE	Chao1	Shannon	Simpson
CK	80,390	7218	14,400	11,570	7.20	2.43E−03
	*10,627*	*568*	*958*	*695*	*0.07*	*4.04E−04*
0.1	78,846	7427	15,211	11,934	7.26	2.17E−03
	*4171*	*306*	*1076*	*591*	*0.02*	*1.15E−04*
0.2	81,497	7422	15,589	12,243	7.15	2.60E−03
	*9060*	*321*	*767*	*607*	*0.06*	*2.0E−04*

**Figure 3:**
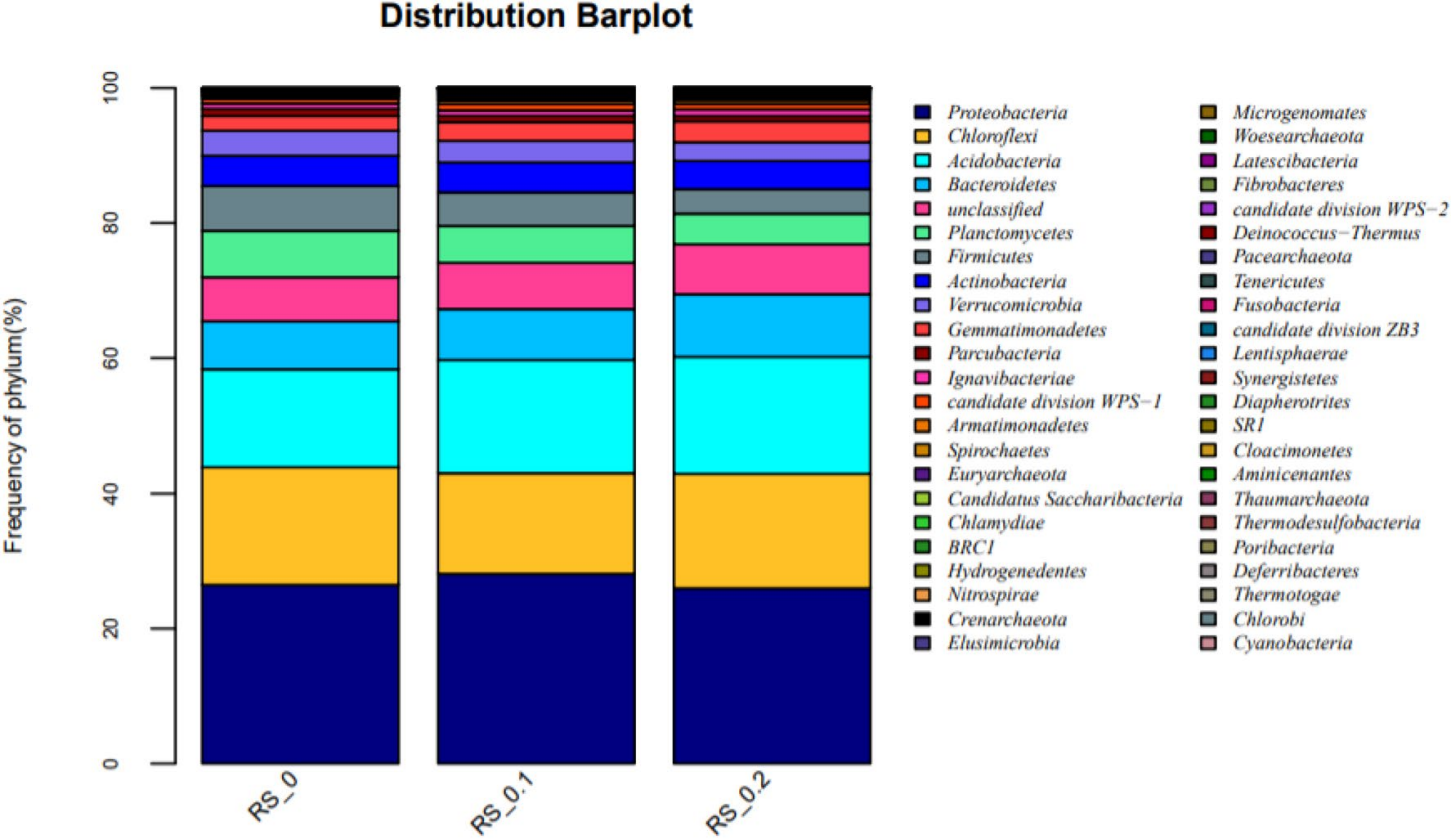
Relative abundance (%) above 1% of different phylum in rhizosphere soil treated with 5 mg/kg Cd under different S fertilization treatments. Others include bacteria with relative abundance < 1%.

The composition of the microbial community in different sulfur treatments and control were further investigated and classified into taxonomic groups. *Proteobacteria*, *Chloroflexi*, *Acidobacteria*, *Bacteroidetes*, *Planctomycetes*, *Firmicutes*, *Actinobacteria and Verrucomicrobia* were dominated phyla (Fig. 3), accounting for 86.93–90.34%. The addition of S^0^ resulted an increase of *Acidobacteria* from 14.48% (CK) to 17.35% (0.2 g/kg) (*P* < 0.05). The ratio of *Bacteroidetes* increased from 7.06% (CK) to 9.26% (0.2 g/kg) (*P* < 0.05). On the other hand, the ratio of *Planctomycetes* decreased from 6.76 to 4.54% (*P* < 0.05) with S^0^ addition (0.2 g/kg). In addition, the ratio of *Chloroflexi* slightly decreased with S^0^ addition.

**Figure 4:**
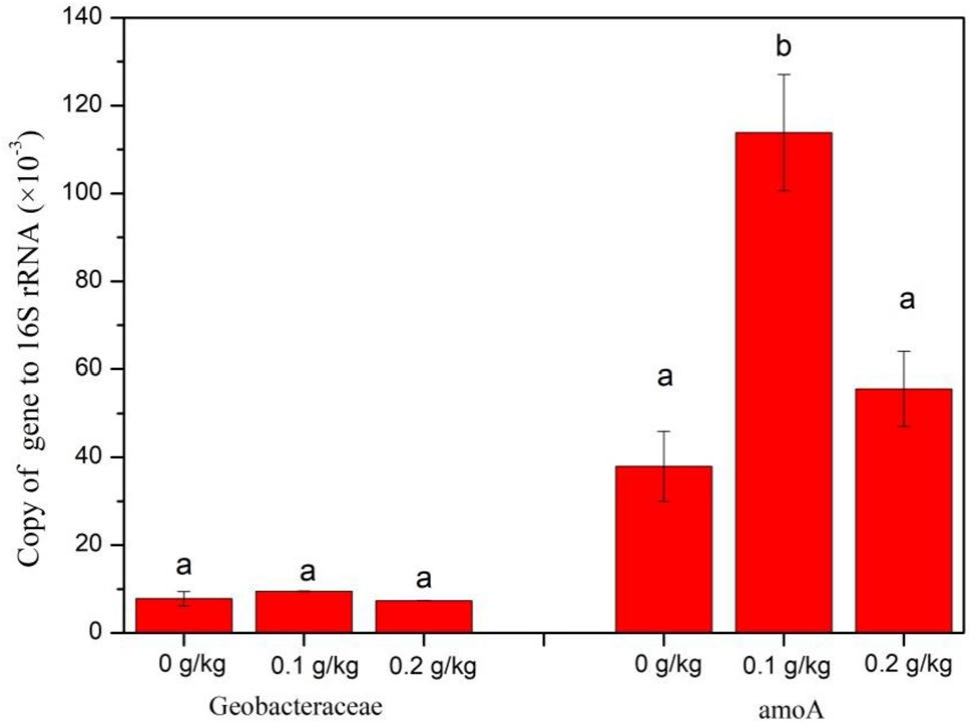
Relative gene abundances of geobacteraceae and amoA in rice rhizosphere soil treated with 5 mg/kg Cd under different sulfur fertilizer treatments. Different capital letters within relative abundances represents a significant difference among different S levels at *P* < 0.05.

In the rhizosphere soil, *Gp6*, *Ornatilinea*, *Anaeromyxobacter*, *Gp3* and *Gemmatimonas* were the most abundant genera in all the treatments. The addition of S^0^ significantly increased the relative abundances of *Gp6* from 2.29% (CK) to 3.04% (0.2 g/kg) (*P* < 0.01), *Gemmatimonas* from 2.18% (CK) to 3.18 (0.2 g/kg), *Gp7* from 1.8% (CK) to 2.5% (0.2 g/kg) (*P* < 0.05) and *Syntrophobacter* from 0.33% (CK) to 0.5% (0.2 g/kg) (*P* < 0.05). Besides, the relative abundance of *Anaeromyxobacter*, *GP3*, showed slight increase with S addition. The addition of S^0^ also had different degrees of inhibitory effects on different genera (Table S1). The relative abundance of *Geobacter* decreased from 0.78% (CK) to 0.57% (0.2 g/kg) (*P* < 0.05), *Bacillus* decreased from 2.23% (CK) to 0.58% (0.2 g/kg) (*P* < 0.05) and *Spartobacteria_genera_incertae_sedis* decreased from 2.01% (CK) to 1.41% (0.2 g/kg) (*P* < 0.05). When compared with the control, the addition of 0.1 g/kg S^0^ significantly increased the relative gene abundances for Geobacteraceae and amoA (Fig. 4).

## Discussion

### Effect of sulfur and Cd on the growth of rice plants

Sulfur is one of the six essential macronutrients in plants, it is widely used to synthesize amino acids and protein^15^. Appropriate application of fertilizer in soil can promote the growth of plants. In the present study, the biomass of rice in soil treated with same Cd was increased S^0^ application, which can be explained by the fertilizer property of sulfur for plants. Besides, Cd is a non-essential element for plants. The exposure of Cd inhibits crop growth and thus reduces their yield through cell proliferation and nitrogen metabolism inhibition as well as photosynthesis rate alteration^16^. In paddy soil under waterlogged condition, higher amount of Cd in soil treated with 5 mg/kg than 1 mg/kg led to the formation more CdS in the same sulfur treatment. The formation of higher amount of CdS may lead to lower amount of bioavailable sulfur in soil, decreasing the amount of sulfur uptake by rice plants and then make the biomass of rice plants less promoted. Moreover, higher amount of Cd addition, like 5 mg/kg, will cause toxicity to rice plants, thus decreasing the biomass of rice plants.

### Cadmium bioavailability and accumulation in rice plants with the addition of sulfur fertilizer

Cadmium is a typical chalcophile element, and it is well known that solubility and bioavailability of Cd in paddy fields decrease when soil is under waterlogged conditions^1,12^. The formation of CdS is the expected mechanism of Cd immobilization in waterlogged paddy soils as the K_sp_ of CdS is 10^–27^, which determines the low solubility of CdS. Hashimoto et al.^17^ found that soil with high sulfur contents showed more rapid removal of Cd from the soil solution and a smaller proportion of Cd in exchangeable fractions when compared with soil with low sulfur contents. The addition of sulfate in paddy soil decreased the Cd:Fe and Cd:Zn ratio in solution during the aerobic phase as up to 34% of sulfur was precipitated as sulfide minerals during the anaerobic phase^18^. In the present study, DGT is applied to determine the bioavailability of Cd. DGT is well known to efficiently simulate the process of plant roots absorbing heavy metals, which can be used for in situ collecting and measuring the bioavailability of heavy metals in soil^19^. The concentrations of DGT-Cd in soil treated with 5 mg/kg Cd decreased with the addition of S^0^, and the decreasing degree is positively associated with the application rate of S^0^. This may be explained by the formation of more proportion of CdS in the rice rhizosphere in S treatment than the control, leading to low content of bioavailable Cd (DGT-Cd) in soil.

The iron plaque forming on rice root surface can adsorb inorganic anions and the efficiency is depended on the amount of iron plaque^20^. The formation of rice root iron plaque is influenced by the content of sulfur in paddy soil; our previous study found that sulfur fertilization (less than 500 mg/kg) promoted the formation of iron plaque, thus sequestering a large amount of Cu on root surface. The moderate and excessive S supply enhanced formation of iron plaque on the rice root surface, which can be one reason for the decreased Cd uptake in rice grains^11^. The addition of Na_2_SO_4_ significantly increased the formation of iron plaque on root surface, while elemental sulfur addition had little effect on iron plaque formation^21^. In our present study, the application of elemental sulfur had no significant effect of iron plaque formation on root surface in soil treated without or with 1 mg/kg Cd. However, in soil treated with 5 mg/kg Cd, application of elemental sulfur significantly increased iron plaque formation and Cd separation. The decreasing of Cd bioavailability in rice rhizosphere soil and increasing of iron plaque formation on root surface lead to lower amount of Cd accumulation in rice tissues.

### Rhizosphere bacterial communities and functional genes with the addition of sulfur fertilizers and their relation to Cd bioavailability

*Proteobacteria*, *Chloroflexi*, *Acidobacteria*, *Bacteroidetes*, *Planctomycetes*, *Firmicutes, Actinobacteria* and *Verrucomicrobia* have been found to be the dominated bacterial in soil, accounting for about 92%^22,23^, which was consistent with our present results accounting for 86.93–90.34% in the rice rhizosphere soil treated with 5 mg/kg Cd. The addition of 0.2 g/kg S^0^ significantly increased the ratio of *Acidobacteria* and *Bacteroidete.* Within the *Acidobacteria, GP3, GP6* and *GP7* were dominated*. GP6 and GP7* were found to be significantly increased by 0.2 g/kg S^0^ addition*. Acidobacteria* is one of the most important bacterial groups in soil; some of them were related to Fe cycling ^24,25^. *Acidobacteria* are commonly classified as slow growing oligotrophys, and dominate in soil with low pH and thrive in soils with relative low available nutrient^26,27^. Wang et al.^28^ reported that some genus belonging to *Acidobacteria* such as *GP6* was a sensitive biomarker that responded only to Cd contamination. The low bioavailable Cd in rice rhizosphere soil in soil treated with 0.2 g/kg S^0^ may be the reason for the increase of *Acidobacteria. Bacteroidete* represents a phylum with ubiquitous distribution, and members of the phylum have the ability to degrade and grow on a variety of complex substrates such as cellulose, chitin and chitin^29^. *Bacteroidete* was reported to be dominant during plant residue decomposition in paddy soil^30^. Higher biomass of rice plants in S^0^ treatments probably provided more substrates for *Bacteroidetes*. Besides, *Bacteroidetes* are widely present in different hypersaline environments and are resistant to salt^31^. The relative abundance of clades within *Bacteroidetes* was significantly increased by the addition of neutral and alkaline salts. In our present study, a trend of increase for soil electricity conductivity (EC) was found with the application of S^0^, which may be another reason for relative higher ration of *Bacteroidetes* in paddy soil treated with S^0^.

*Syntrophobacter* is a genus that belongs to *Syntrophobacteraceae*, which is associated with sulfate reduction. It has been reported that the addition of gypsum in paddy soil increased the relative abundance of *Syntrophobacteraceae* (*Desulfovirga* spp., *Syntrophobacter* spp. and unclassified *Syntrophobacteraceae*)^32^. Chen^33^ also reported that the addition of Na_2_SO_4_ increased the abundance of *Syntrophobacteraceae*, leading to reduction of sulfate to S^2−^. Our present result is in consistent with these studies, the addition of S^0^ led to the significantly increase of *Syntrophobacter*, which means the addition of S^0^ promoted the reduction of sulfate to S^2−^. The promoted formation of S^2−^ led to formation of CdS, thus reducing the bioavailability of Cd. Geobacter is a genus that belong to *Deltaproteobacteria*, which is commonly associated with Fe (III)-reduction. In our present study, the addition of 0.1 g/kg S^0^ showed a trend of increase for the relative gene abundances of geobacteraceae, which means that the addition of 0.1 g/kg S^0^ may increase the process of iron cycling. In paddy soil with flooded treatments, the abundance of *Geobacter* was found higher than those with non-flooded and alternate wetting and drying treatments^34^. Tang et al.^21^ found that the genera belonging to *Deltaproteobacteria* (including *Anaeromyxobacter, Geobacter*, *Syntrophorhabdus*, *Deferrisoma*, and *Syntrophus*) were significantly increased with the addition of Na_2_SO_4_. In our present study, the abundance of *Geobacter* was significantly decreased by the addition of 0.2 g/kg S^0^.

## Conclusion

The influence of sulfur fertilizer on Cd biogeochemical behavior in soil, accumulation in rice plants and microbial community in soil was investigated in the present study. The addition of S^0^ increased the biomass of rice plants by 19.5–73.6%. The addition of S^0^ increased the formation of iron plaque by 15.3–31.3%, meanwhile the amount of Cd sequestered on iron plaque increased. In soil treated with 5 mg/kg Cd, the bioavailability of Cd decreased by 60.0% with the addition of 0.2 g/kg S^0^. The application of S^0^ significantly decreased the concentration of Cd in rice grain by 12.1% (0.1 g/kg) and 36.6% (0.2 g/kg) respectively. The addition of S^0^ significantly increased the ratio of *Acidobacteria*, *Bacteroidetes* in soil rice rhizosphere soil treated with 5 mg/kg Cd. Meanwhile, the ratio of *Planctomycetes* and *Chloroflexi* decreased. Our results indicated that S^0^ can be a potential pathway to reduce Cd migration from paddy soil to food chain.

## Materials and methods

### Experimental design

The soil was collected from the top layer (0–20 cm) of paddy field in Changsha city, Hunan province, China. After air-drying, the paddy soil was ground to particle size of less than 2 mm. The basic physical and chemical properties of the tested soil can be referred in Table S1. Ground soil was mixed with 0, 1 or 5 mg/kg Cd^2+^ (in the form of CdCl_2_ solution) respectively. The CdCl_2_ solution with different Cd concentration was prayed thoroughly to soil, and a glass bar was used to stir the soil to mix soil with solution. The soil mixed with Cd^2+^ was then put into a dark environment with controlled temperature and humidity condition. After aged with deionized water to keep 30% moisture content for 30 days, contaminated soil was used for pot experiment.

Elemental sulfur was mixed thoroughly with soil, and each treatment was replicated three times. In order to provide basal fertilizer, chemical fertilizer was applied as the following details: phosphorous was applied as Ca(H_2_PO_4_)_2_H_2_O at 0.15 g P_2_O_5_/kg, K as KCl at 0.2 g K_2_O/kg, and N as urea at 0.2 g N/kg; fertilizers were mixed thoroughly with soil at the beginning of the experiment. Element sulfur was applied at three rates (0, 100, and 200 mg/kg)^10^. A rhizobag (size 13 cm in height × 6 cm in diameter; 300 μm) was put in a plastic drum (size 13 cm in height × 12 cm in diameter) and the rhizosphere soil was defined as the one in the rhizobag near rice root. As designed, 2 kg of sieved soil was added to the plastic drum, with 1 kg in the rhizobag and 1 kg outside in the drum.

The conventional rice (*Oryza sativa* L.) seeds (Cultivar “Zhongzao 39”, accession number: 2012015, purchased from Wuwangnong Group Co., Ltd, China) was chosen in this experiment as it is widely grown in Yangtze river delta, China. Plant experiments were carried out in accordance with relevant guidelines. After being surface-sterilized in 30% (v/v) hydrogen peroxide (H_2_O_2_) solution for 15 min, the rice seeds were thoroughly washed with deionized water. The seeds were germinated on moist gauze mounted on a nylon fine screen and immersed in deionized water. Ten days later, rice seedlings were transplanted into nutrient solution as suggested by the International Rice Research Institute. Two weeks later, two seedlings were transplanted into the rhizobag. Distilled water was added each day to keep flooding conditions with a layer of water of 4–5 cm above the soil surface. All pots were placed in a growth chamber randomly with 60–70% relative humidity, day/night duration 16/8 h, and day/night temperature 25/20 °C^35^. The redox potential (Eh) values were detected in situ by inserting an oxidation reduction electrode into the rhizosphere soil at the same depth (about 3 cm).

### Harvest and sampling

Three months after germination, rice plants were harvested and washed gently with distilled water thoroughly. Plants were separated into leaves, stems and roots by stainless steel scissors. The rhizosphere soil was collected from the rhizobag by a wooden spoon. All the samples were freeze-dried before analysis. Dried plant tissues were milled into homogenized powders by a grinding miller (Tissuelyser-24, China) after weighed and then stored in a vacuum drier before analysis.

### Cd concentration in rice plants

Samples were digested with concentrated nitric acid-30% H_2_O_2_-HF in the microwave digestion apparatus (CEM Mars One, USA). Total amount of heavy metals in the digestion solution was determined by graphite furnace atomic absorption spectrometry (GFAAS) (Agilent, USA).

### Cd concentration in rhizosphere soil extracted by diffusive gradients in thin films (DGT)

The DGT device consists of a diffusive layer with polyacrylamide and DGT crosslinker overlying a restricted gel layer containing Chelex-100 resin, a dialysis membrane, as well as plastics molding. The cylindrical DGT devices (Chelex DGT) used in the present study were purchased from Easy Sensor (Nanjing, China). The freeze-dried paddy soil samples were analyzed by DGT according to the method described in the supplementary material.

### Root iron plaque extraction

At harvest, iron plaque on fresh rice root surface were extracted by ascorbic citrate acetic (ACA) solution, which contained 0.3 M sodium citrate (40 mL), 10% sodium acetic (5 mL), and 3 g ascorbic acid^36^. Roots of rice were immersed for 3 h at 25 °C in 45 mL of ACA solution. Roots were then rinsed three times with distilled water, collecting the water in the ACA extracts^36^. The final volume of the extracting solution was made up to 100 mL using deionized water. After filtration with quantitative filter papers to remove the small debris roots, the solution was stored at 4 °C before analysis. Cd and Fe concentration in the extraction was determined by ICP-MS (7900, Agilent, USA).

### DNA extraction and PCR amplification

DNA was isolated from the fresh rhizosphere soil using the E.Z.N.A™ Mag-Bind Soil DNA Kit (OMEGA, USA), according to the manufacturer’s instructions^20^. The V3-V4 region of the bacterial 16 S rRNA gene was amplified with the universal primers 341F (CCCTACACGACGCTCTTCCGATCTG) and 805R (GACTGGAGTTCCTTGGCACCCGAGAATTCCA). The PCR conditions according to Tang with some appropriate modifications, each 30 μL PCR mixture contained 10–20 ng DNA. The cycling conditions included a pre-denaturation at 94 °C with 3 min, denaturation at 94 °C for 30 s, annealing at 45 °C for 20 s, 5 cycles of extension at 65 °C for 30 s, annealing at 55 °C for 20 s, 20 cycles of extension at 72 °C for 30 s and a final extension was performed at 72 °C for 5 min. For subsequent sequencing, the DNA was accurately quantified using the Qubit 3.0 DNA Assay Kit (Life, USA). The obtained raw sequence data, containing linker and barcode sequence, had a certain amount of interference data, these reads were removed and then the overlapped reads were spliced using the PEAR software. The spliced data were filtered using the QIIME program, the N-containing or low-mass sequences were filtered out. Finally, the chimeric sequences were removed and the resulting sequences obtained clustered into operational taxonomic units (OTUs) by UPSRSE. Representative sequences from each OTUs were aligned with SILVA databases.

### Functional genes

Bacterial populations involved in the cycles of Fe, S and N were detected by quantitative real-time PCR. The absolute gene copy numbers of the target genes were normalized to that of the 16S rRNA genes, to minimize variances caused by different background bacteria and analytical efficiencies and expressed as the relative gene abundances (Fig. 4).

### Data analysis

Analysis of one-way ANOVA on plant biomass and Cd concentration in DGT-extractable Cd and two-way ANOVA on Cd concentration in rice tissues and ACA-extractable Fe and Cd were performed using Windows-based SPPS 20.0 (SPSS Inc. USA). Data are represented as mean value ± standard deviation (SD) (three treatments) in all figures. The statistical significance (*P* < 0.05) of differences among values in the treated samples and the controls was evaluated by LSD test. All the figures were made by Origin Pro 8.0.

## Supplementary Information

Supplementary Information.
