# Supplementary material for: Influence of elemental sulfur on cadmium bioavailability, microbial community in paddy soil and Cd accumulation in rice plants
Source: Sci Rep. 2021 Jun 1;11:11468. doi: 10.1038/s41598-021-91003-x (PMC8169911; doi:10.1038/s41598-021-91003-x)
Supplement: Supplementary file 1 — Supplementary Information. [file 41598_2021_91003_MOESM1_ESM.docx]

Influence of elemental sulfur on cadmium bioavailability, microbial community in paddy soil and Cd accumulation in rice plants

Lijuan Sun^1, 2^, Ke Song^1, 2^, Lizheng Shi ^3^, Dechao Duan^4^, Hong Zhang^1, 2^, Yafei Sun^1, 2^, Qin Qin^1, 2^, Yong Xue^1, 2 *^

^1^ ECO-Environment Protection Research Institute, Shanghai Academy of Agricultural Sciences, Shanghai, 201403

^2^ Shanghai Environmental Protection Monitoring Station of Agriculture, Shanghai, 201403

^3^ South China Institute of Environmental Sciences, Ministry of Ecology and Environmental, No. 16-18, Ruihe Road, Huangpu District, Guangzhou, 510530, Guangdong

^4^ Zhejiang Towards Environment Co., Ltd, Hangzhou 310012, China

* Corresponding author

(Yong Xue) [exueyong@163.com](mailto:exueyong@163.com)

Table S1 Basic physical and chemical properties of the tested soil

| Tested soil | pH | Organic matter  (g/kg) | Electric conductivity  (mS/cm) | Total Fe  (g/kg) | Total P  (g/kg) | Total K  (g/kg) | Total Cd  (mg/kg) | Available Cd  (mg/kg) |
| --- | --- | --- | --- | --- | --- | --- | --- | --- |
| Paddy soil | 6.43 | 12.25 | 0.35 | 37.52 | 0.74 | 21.51 | 0.17 | None detected |

**Table S2** The responses of genera in rhizosphere soil to different sulfur fertilizers.

| **Response** | **Genus in rhizosphere soil** | **Significant difference** | |  |
| --- | --- | --- | --- | --- |
|  |  | 0.1 g/kg S | 0.2 g/kg S |  |
| Promoted | *GP6* |  | ** |  |
|  | *GP3* | * | * |  |
|  | *Gemmatimonas* |  | * |  |
|  | *GP7* |  | * |  |
|  | *Tepidisphaera* | * | * |  |
|  | *Syntrophobacter* |  | * |  |
|  | *Ralstonia* |  | ** |  |
| Inhibited | *Geobacter* | ** | * |  |
|  | *Bacillus* | * | * |  |
|  | *Spartobacteria_genera_incertae_sedis* |  | * |  |

The asterisk indicates significant differences between control (CK) and other treatments. * *p* < 0.05, ** *p* < 0.01, *** *p* < 0.001.

**Cd in the rhizosphere Soil Extracted DGT**

A total amount of 10 g soil samples were placed in clean beaker, added with Milli-Q water to obtain 70% maximum field water holding rate, mixed thoroughly with a glass bar and equilibrated at 25 ± 1 ℃ for 48 h. A plastic wrap was kept on the beaker to avoid the evaporation of water. About 3 g soil was then put into the DGT units by a clean plastic spoon and shaken gently on the desk to make soil fully exposed to the filter membrane. DGT units with soil was maintained for 24 h at 25 ± 1 ℃, then were removed from the soil and washed with Milli-Q water. The resin gel was immersed in 1 mol/L HNO_3_ for 24 h and the metal contents were measured by ICP-MS. The effective concentration of DGT (C_DGT_) was calculated according to the following formula (1) ([Zhang and Davison, 1995](#_ENREF_5); [Zhang et al., 1998](#_ENREF_6)).

$C_{DGT}=\frac{C_{e}(V_{e}+V_{g})\cdot\triangle g}{D\cdot A\cdot t\cdot f_{e}}$ （1）

where *C_e_* is the concentration of metals in the elution solution, *V_e_* is the volume of HNO_3_ added to the resin gel (1.8 mL), *V_g_* is the volume of the resin gel (0.2 mL), *Δg* is the thickness of diffusive layer (cm); *D* is the [diffusion coefficient](https://www.sciencedirect.com/topics/earth-and-planetary-sciences/diffusion-coefficient) of metallic element in the diffusive layer (cm^2^·s^−1^); *A* is the area of DGT exposure window (3.14 cm^2^); and *t* is the DGT deployment time (s), and *f_e_* is the elution factor for the metals (0.938).

**
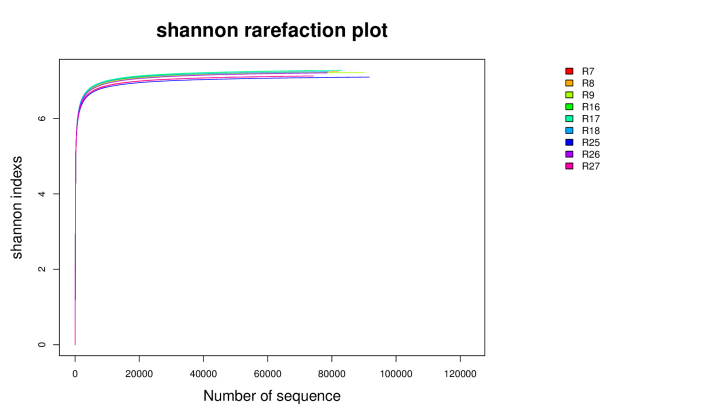
**

**
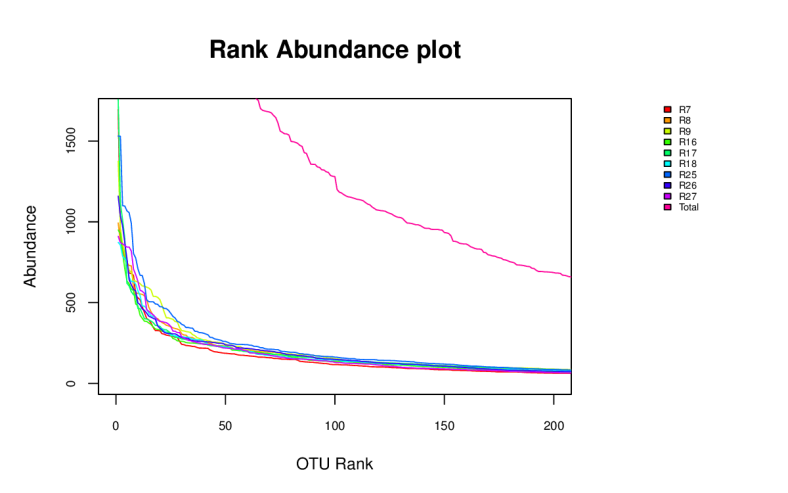
Figure S1** Rarefaction curves (A) and Rank abundance (B) for bacterial OTUs in rhizosphere soil treated with 5 mg/kg Cd under different S fertilization treatments: R7-R9: CK, R16-18: 0.1 g/kg S^0^; R25-R27：0.2 g/kg S^0^
